# High-Temperature Short-Time and Holder Pasteurization of Donor Milk: Impact on Milk Composition

**DOI:** 10.3390/life11020114

**Published:** 2021-02-03

**Authors:** Diana Escuder-Vieco, Juan M. Rodríguez, Irene Espinosa-Martos, Nieves Corzo, Antonia Montilla, Alba García-Serrano, M. Visitación Calvo, Javier Fontecha, José Serrano, Leónides Fernández, Carmen Rosa Pallás-Alonso

**Affiliations:** 1 Banco Regional de Leche Materna, Hospital Universitario 12 de Octubre, Instituto de Investigación i+12, 28041 Madrid, Spain; 2 Sección Departamental de Nutrición y Ciencia de los Alimentos (Veterinaria), Universidad Complutense de Madrid, 28040 Madrid, Spain; jmrodrig@ucm.es; 3 Probisearch S.L., Tres Cantos, 28760 Madrid, Spain; irenee70@gmail.com; 4 Departamento de Bioactividad y Análisis de Alimentos, Instituto de Investigación en Ciencias de la Alimentación, CIAL (CSIC-UAM), 28049 Madrid, Spain; nieves.corzo@csic.es (N.C.); a.montilla@csic.es (A.M.); albamaria.garcia.serrano@csic.es (A.G.-S.); mv.calvo@csic.es (M.V.C.); j.fontecha@csic.es (J.F.); 5 Departament de Medicina Experimental, Facultad de Medicina, Universitat de Lleida, 25008 Lleida, Spain; jcserrano@mex.udl.cat; 6 Sección Departamental de Farmacia Galénica y Tecnología Alimentaria (Veterinaria), Universidad Complutense de Madrid, 28040 Madrid, Spain; 7 Servicio de Neonatología, Hospital Universitario 12 de Octubre, Instituto de Investigación i+12, Universidad Complutense de Madrid, 28041 Madrid, Spain; kpallas.hdoc@gmail.com

**Keywords:** donor milk, preterm nutrition, HTST pasteurization, holder pasteurization, macronutrients, *myo*-inositol, fatty acids, bile salt stimulated-lipase, vitamins

## Abstract

Holder pasteurization (HoP; 62.5 °C, 30 min) is commonly used to ensure the microbiological safety of donor human milk (DHM) but diminishes its nutritional properties. A high-temperature short-time (HTST) system was designed as an alternative for human milk banks. The objective of this study was to evaluate the effect of this HTST system on different nutrients and the bile salt stimulated lipase (BSSL) activity of DHM. DHM was processed in the HTST system and by standard HoP. Macronutrients were measured with a mid-infrared analyzer. Lactose, glucose, *myo*-inositol, vitamins and lipids were assayed using chromatographic techniques. BSSL activity was determined using a kit. The duration of HTST treatment had a greater influence on the nutrient composition of DHM than did the tested temperature. The lactose concentration and the percentage of phospholipids and PUFAs were higher in HTST-treated than in raw DHM, while the fat concentration and the percentage of monoacylglycerides and SFAs were lower. Other nutrients did not change after HTST processing. The retained BSSL activity was higher after short HTST treatment than that following HoP. Overall, HTST treatment resulted in better preservation of the nutritional quality of DHM than HoP because relevant thermosensitive components (phospholipids, PUFAs, and BSSL) were less affected.

## 1. Introduction

The composition of human milk is dynamic and depends on different factors, including host genetics, environment and gestational age [1]. As a result, own mother’s milk (OMM) is widely acknowledged as the best feeding option for preterm infants [2]. However, milk production by mothers of very preterm infants is frequently delayed and/or insufficient during the first days after birth, and some initiatives to achieve an adequate milk supply for the infant, such as the use of galactogogues, have proved to be unsuccessful [3]. When OMM is unavailable or in short supply for meeting the nutritional requirements of preterm infants, which is common in neonatal intensive care units, the best alternative is the use of donor human milk (DHM) [4]. DHM pasteurization at 62.5 °C for 30 min (Holder pasteurization; HoP) is the most commonly used treatment in human milk banks (HMB) to ensure its microbiological safety. Unfortunately, this heating treatment has a negative impact on some of the nutritional and bioactive properties of human milk [5,6].

Human milk carbohydrates, including lactose and oligosaccharides, and lipids, such as arachidonic (ARA) and docosahexaenoic (DHA) acids, appear to be unaffected by HoP [7,8]. In contrast, this treatment reduces the biological activity of some proteins, including lactoferrin and lysozyme [9]. Moreover, the concentration and activity of bile salt-stimulated lipase (BSSL), which is involved in the digestion of milk triglycerides in breastfed infants, is lost after HoP [10]. In relation to DHM vitamins, the results obtained so far on the impact of HoP are largely inconclusive. It seems that the levels of some water-soluble vitamins, such as vitamin C, folate or B6, were lower after this treatment, while those of vitamins A, D and E remained unaffected [6,11].

Understanding the potential impact of pasteurization on milk components provides an important tool for the management of infant feeding, particularly to meet the nutritional requirements of preterm infants according to their body weight. Recently, high-temperature short-time (HTST; 72 °C, 15 s) pasteurization has been proposed as an alternative treatment for DHM [4,12]. Different experimental systems, such as laboratory capillary heat exchangers, industrial heat exchangers or benchtop devices, have been designed for HTST pasteurization of DHM [12,13,14] but they have never been tested under authentic HMB conditions.

Recently, a continuous HTST system that was developed to pasteurize DHM in the HMB-operating environment was designed and validated [15]. This new system ensures the microbiological safety of DHM with minimal heat damage and preserves some bioactive factors, including immunoglobulins, growth factors and hormones, after DHM treatment at 72 °C for at least 10 s [15,16]. Accordingly, the objective of this study was to evaluate the impact of this new HTST system compared to standard HoP on the DHM content of macronutrients, glucose, *myo*-inositol, selected water-soluble vitamins (thiamine, riboflavin, FAD, vitamin B2, nicotinamide, pyridoxal and cyanocobalamin) and fat-soluble vitamins (vitamin A, α-tocopherol, γ-tocopherol, vitamin D_3_ and vitamin 25(OH)D_3_), as well as the fatty acid (FA) profile and BSSL activity.

## 2. Materials and Methods

### 2.1. Human Milk Samples

DHM samples were obtained from 48 donor mothers of the Regional Human Milk Bank “Aladina-MGU” (Hospital Universitario 12 de Octubre, Madrid, Spain). Mean (SD) age of donors was 33.4 (3.8) years. Among these donor mothers, 80% had a term delivery and 62% were primigravida. A total of 90% of women delivered vaginally, and the mean gestational age of their children was 37.1 (5.8) weeks. All donors gave samples of mature milk (more than 15 days after birth).

Milk collection was performed following a specific protocol for donor mothers, which was approved by the Hospital 12 de Octubre Clinical Research Ethics Committee (ethical approval code: 12/325). Informed consent was obtained from each participating donor in accordance with the Declaration of Helsinki. Milk was collected at home, frozen (−18 °C) afterwards in a domestic freezer, and then transported to the HMB in an insulated box with provided ice packs.

### 2.2. Experimental Design

A total of 10 DHM production batches were used in this study. Each production batch (10 L) was composed of milk from approximately 12 donors (Figure S1). A 120 mL aliquot of each production batch was kept to be used as a control (raw milk). Another 120 mL aliquot was subjected to HoP (62.5 °C for 30 min) and fast cooling at 4 °C in shaking water baths (Jeio Tech BS-21, Lab Companion, Oxfordshire, UK) following the standard HMB procedure. The rest of the batch was processed at a fixed temperature (70, 72 or 75 °C) for different times (from 5 to 25 s) using the HTST equipment previously described by Escuder et al. [15]. Three production batches were processed at 70°C, 4 batches at 72 °C and 3 batches at 75 °C. Some aliquots of the raw and heat-treated DHM were used immediately after treatment to determine the macronutrient content, while the rest of them were stored at −20 °C until further analysis was performed. Aliquots used for light-sensitive vitamin quantification were stored in black microcentrifuge tubes (LTT-170-X; Mettler Toledo, L’Hospitalet de Llobregat, Barcelona, Spain).

### 2.3. Analysis of Macronutrients

Total fat, protein and lactose in raw and heat-treated DHM were measured in duplicate by Fourier-transform mid-infrared (FT-MID) spectroscopy in a milk analyzer (MilkoScan FT2, FOSS S.A., Barcelona, Spain) properly calibrated for the analysis of human milk.

### 2.4. Determination of Lactose, Glucose and myo-Inositol

Lactose, glucose, and *myo*-inositol analysis was carried out by gas chromatography (GC) with flame ionization detection following the method described by Montilla et al. [17] with the modifications described by Espinosa-Martos et al. [18]. The identity of the carbohydrates present in DHM samples was confirmed by relative retention time comparison with those of standard samples. Quantitative analysis was achieved with the internal standard method. Response factors were calculated after triplicate analysis of standard solutions (glucose, *myo*-inositol and lactose) at concentrations ranging from 1 to 6 g/L (lactose) or from 1 to 50 mg/L (glucose and *myo*-inositol). Analyses of milk samples were performed in duplicate.

### 2.5. Analysis of Lipids

Total fat extraction in DHM samples was achieved using a modification of the Folch method [19] and a dichloromethane-methanol solution (2:1 *v*/*v*) as the lipid solvent. The extract was concentrated by removing dichloromethane in a rotatory evaporator and dried under a gentle nitrogen stream. The extracted fat was weighed in amber vials flushed with nitrogen and stored at −35 °C until chromatographic analysis.

Separation and quantification of lipid classes was performed by high-performance liquid chromatography (HPLC) (Agilent Technologies, model 1200; Agilent Technologies Inc., Palo Alto, CA, USA) with evaporative light scattering detection (SEDERE SEDEX model 85, Alfortville Cedex, France). Prefiltered compressed air was used as the nebulizing gas at 350 kPa and 90 °C, while the gain was set at 6. Two Zorvax Rx-SIL columns (250 mm × 4.5 mm and 5 μm particle size, Agilent Technologies Inc.) were coupled in series with a precolumn with the same stationary phase and were equilibrated at 40 °C. The injection volume was 50 μL at a concentration of 5 mg/mL in methylene chloride. The solvent gradient program has been described previously [19]. Lipid standards were used for the identification of lipid classes. Assays were carried out in triplicate.

FAs from the DHM samples were directly derivatized to FA methyl esters (FAMEs) according to Castro-Gómez et al. [19]. FAMEs were analyzed using an Agilent 6890N GC system (Agilent Technologies Inc.) equipped with a flame ionization detector connected to a 5973N quadrupole mass selective detector (Agilent Technologies Inc.). Chromatographic separation was performed on a CP-Sil 88 fused-silica capillary column (100 m × 0.25 mm i.d. × 0.2 µm film thickness; Chrompack, Middelburg, The Netherlands) as described previously [20]. The MS detector was operated in electron impact mode at 70 eV, the transfer line temperature was set at 250 °C, the ion source was set at 230 °C, and the quadrupole temperature at 150 °C; the scan was set to obtain a mass spectrum over a mass range of 50–500 Da. The sample volume injected was 1 µL at a 1:25 split ratio. Anhydrous milk fat (reference material BCR-164; Fedelco Inc., Madrid, Spain) was assayed to determine and calculate the response factor for FAMEs, while glyceryl tritridecanoate (100 µL of 1.28 mg/mL) was used as an internal standard. Assays were carried out in triplicate.

### 2.6. Determination of BSSL Activity

BSSL activity in the DHM samples was determined using a lipase activity assay kit (Sigma-Aldrich Química S.L., Madrid, Spain) following the manufacturer’s instructions. Sodium taurocholate (10 mM) was added to the lipase assay buffer. After 100 min at 37 °C, the absorbance was determined at 570 nm using a Zenyth 200 microplate reader and spectrophotometer (Anthos Labtec, Salzburg, Austria). One unit of lipase activity was defined as the amount of enzyme that would generate 1.0 µmole of glycerol from triacylglycerides (TGs) per minute at 37 °C.

### 2.7. Analysis of Vitamins

The analysis of water-soluble vitamins (thiamine, riboflavin, flavin adenine dinucleotide (FAD), nicotinamide (vitamin B_3_) and pyridoxal) was carried out by ultra-performance liquid chromatography−tandem mass spectrometry (UPLC–MS/MS) following the method described by Hampel et al. [21]. Prior to the analysis, samples were subjected to protein precipitation and removal of nonpolar constituents by diethyl ether. Quantification was performed by ratio response to the isotope-labeled internal standards.

Cyanocobalamin was determined by UPLC–MS/following the procedure described by Zhang et al. [22]. Briefly, samples were centrifuged to separate and eliminate fat. The extraction procedure was conducted with aqueous sodium acetate while 150 µL of 1% potassium cyanidin and methotrexate was used as an internal standard. The samples were heated at 90 °C to ensure quantitative conversion of all forms of vitamin B_12_ to cyanocobalamin.

The concentrations of retinol (the main form of vitamin A in milk), α-tocopherol (the main biological form of vitamin E), and γ-tocopherol were determined via HPLC with fluorescence and ultraviolet detection following the method described by Jiang et al. [23]. The concentration of tocopherols was determined with an excitation wavelength of 295 nm and a cutoff emission filter of 345 nm, and retinol was determined by ultraviolet detection (at 325 nm). External quantification was performed based on calibration curves for retinol and α-tocopherol. Briefly, samples were saponified with a mixed solution of 0.1 g of ascorbic acid, 2 mL of ethanol including 0.1% butylated hydroxytoluene and 0.5 mL of 50% aqueous potassium hydroxide solution. Subsequently, retinol and tocopherols were extracted with petroleum ether and the organic fraction was dried with nitrogen and later dissolved with a mixed solution of methanol and methyl *tert*-butyl-ether (1:1 *v*/*v*) including 0.1% butylated hydroxytoluene.

Vitamin D_3_ and vitamin 25(OH)D_3_ were determined by UPLC–electrospray ionization/tandem MS as described previously [24]. After protein precipitation with acetonitrile, vitamin D metabolites were extracted with methyl *tert*-butyl-ether and the organic fraction was dried under nitrogen. Furthermore, the samples were dissolved with methanol and 4-phenyl-1,2,4-triazoline-3,5-dione for derivatization. Deuterated metabolites of vitamin D were used as internal standards during the whole process.

### 2.8. Statistical Analysis

The normality of the data distribution was tested through histograms and Shapiro–Wilk tests. Data are presented as the means and SEM. The enzyme activity of BSSL in heat-treated samples was expressed as the remaining activity in relation to that detected in raw DHM samples. Repeated measures two-way ANOVA was used to test the effect of the main variables (time and temperature) of HTST treatment on the different milk components and BSSL activity (PROC MIXED with REPEATED statement of the SAS System with restricted maximum-likelihood estimation, including fixed effects for the duration (time) and the temperature (temperature) of each HTST treatment, and their interaction). Data were grouped by the duration of HTST treatment to compare the influence of HTST treatment and HoP on the nutrient composition and residual BSSL activity in relation to those of raw DHM. For these comparisons, data were analyzed using repeated measures one-way ANOVA tests including treatment (raw, HTST at different treatment durations and HoP) as fixed effects. Dunnett’s post hoc tests were performed to verify the significance of differences at the 95% confidence level in pairwise comparisons of the mean nutrient concentration in heat-treated (HTST at different treatment durations and HoP) DHM with that of the raw control DHM (a total of six pairwise comparisons). Pearson’s correlation coefficient test was applied to compare the lactose concentration values obtained by mid-IR analysis (milk analyzer) and GC. The statistical software Statgraphics Centurion XVI version 17.0.16 (Statpoint Technologies Inc., Warrenton, VA, USA) and SAS 9.4 (SAS Institute Inc., Cary, NC, USA) were used to perform these analyses.

## 3. Results

### 3.1. Macronutrients

**Figure 1:**
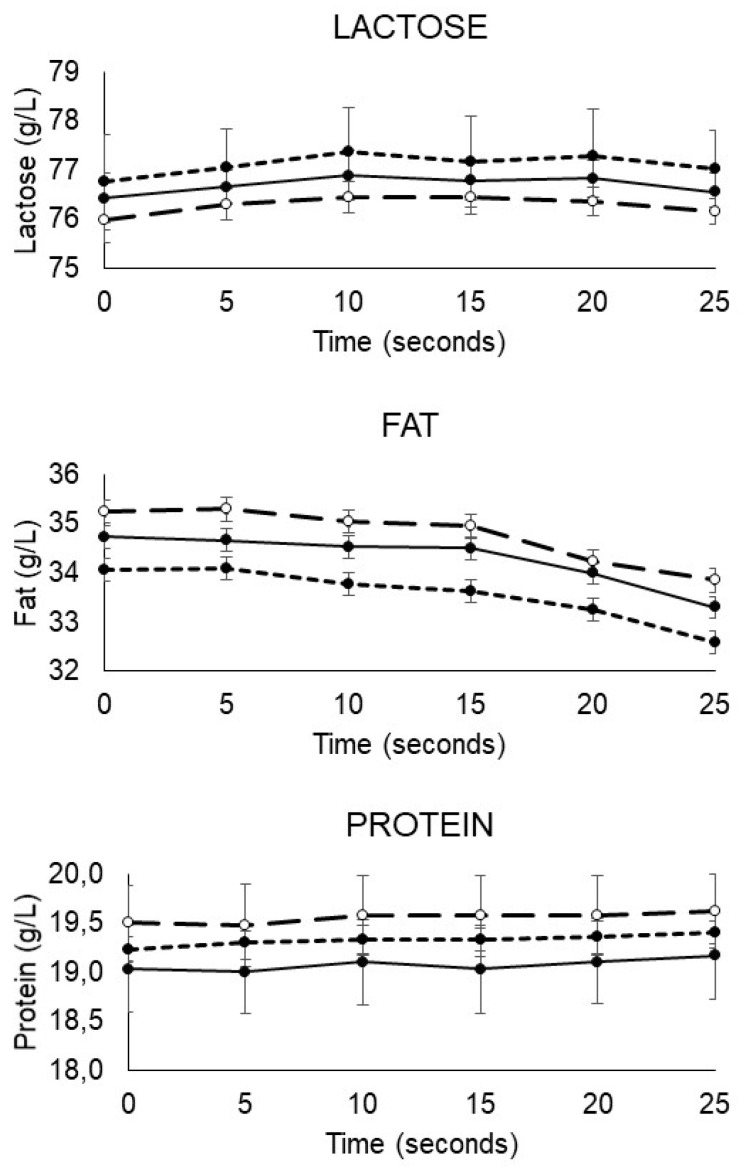
Concentration of macronutrients (lactose, fat, and protein) in 10 batches of donor milk after HTST treatment at 70 °C (○, dashed line; *n* = 3), 72 °C (●, dashed line; *n* = 4), and 75 °C (●, solid line; *n* = 3). Samples were regularly taken at 0 (raw milk), 5, 10, 15, 20, and 25 s. Repeated measures two-way ANOVA tests were used to determine the impact of temperature and duration of HTST treatment, and their interaction on the concentrations of lactose, fat, and protein. There was no interaction between the two factors, and only the duration of HTST treatment had a statistically significant effect on the concentration of lactose and fat (*p* < 0.001).

Lactose was the most widely available macronutrient fraction in DHM. The mean (SEM) lactose concentration in the DHM samples obtained from the unprocessed batches was 76.4 (0.3) g/L when measured by FT-MID spectroscopy. The mean (SEM) values for fat and protein were 34.7 (0.7) g/L and 19.3 (0.2) g/L, respectively (Figure 1). In raw DHM, a lower intersample variability was observed for protein (IQR = 4.7 g/L) and lactose (IQR = 4.1 g/L) than for fat (IQR = 8.8 g/L).

HTST processing of DHM led to minor but statistically significant changes in the lactose and fat concentrations but not in the protein concentration. These changes were related to the duration of the HTST treatment rather than to the tested temperatures (repeated measures two-way ANOVA tests, *p* < 0.001 for the effect of duration of the HTST treatment on lactose and fat concentrations) (Figure 1). The impact of the duration of the HTST treatment followed the same trend among the different temperatures tested in the present study (70 °C, 72 °C, or 75 °C), as indicated by the lack of interaction between time and temperature for each HTST treatment.

Variations in the macronutrient composition of DHM after HTST treatments and HoP are shown in F. The lactose concentration was higher (between 0.2 and 0.5 g/L) in HTST-treated samples than in raw DHM (repeated measures one-way ANOVA, *p* < 0.001), except for in samples subjected to the longest treatment (25 s). The fat content after HTST treatments for 5–15 s did not differ from that of raw DHM, but after the longer treatments (20 and 25 s), the DHM fat levels were lower (0.8 and 1.4 g/L, respectively) than in unprocessed DHM (repeated measures one-way ANOVA, *p* < 0.001). Additionally, the protein concentration did not vary with any of the HTST treatments compared to that of raw DHM. Furthermore, with respect to those in unprocessed DHM, there were no differences in lactose and fat contents, but the protein level was lower (0.2 g/L) after HoP (repeated measures one-way ANOVA, *p* < 0.001) (Table 1).

**Figure 2:**
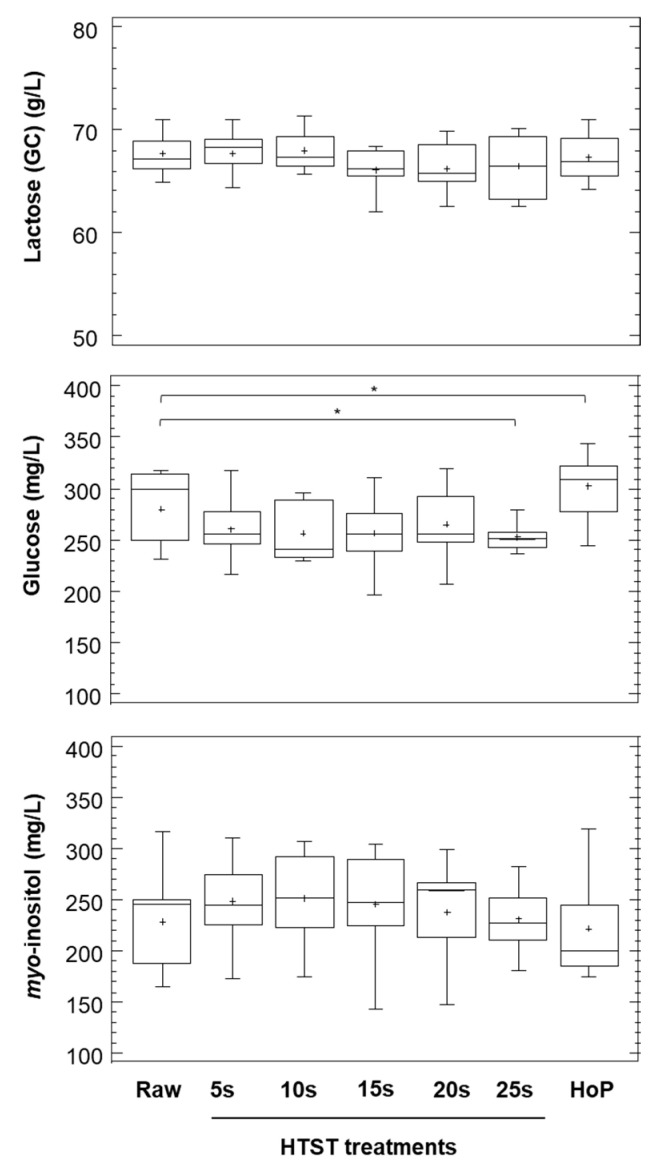
Concentrations of lactose, glucose and *myo*-inositol in DHM before (raw) and after HTST treatment for 5 (5 s), 10 (10 s), 15 (15 s), 20 (20 s) and 25 (25 s) seconds or HoP (62.5 °C, 30 min; HoP) (*n* = 10). Lactose, glucose, and *myo*-inositol concentrations were determined by GC. The height of the box indicates the IQR, the horizontal line in the box represents the median concentration value and the cross represents the mean concentration value. Data were grouped according to the duration of the HTST treatment before being analyzed using repeated measures one-way ANOVA including treatment (raw, HTST with different dura-tion of treatment and HoP) as fixed effects. * Asterisks indicate significant differences in pairwise comparisons between raw and heat-treated (HTST treatment for different durations or Holder pasteurized) DHM (post hoc Dunnett’s tests at the 95% confidence level). DHM, donor human milk; HoP, Holder pasteurization; HTST, high-temperature short-time.

The mean (SEM) lactose concentration in raw DHM samples (measured using GC) was 67.8 (0.7) g/L, and this value was similar across every HTST treatment and HoP (Figure 2; Table S1). The correlation between the lactose concentration measured by FT-MID spectroscopy (milk analyzer), which also includes oligosaccharides, and the lactose concentration determined by the chromatographic method was weak (r = 0.324, *p* = 0.025).

### 3.2. Glucose and myo-Inositol

Other carbohydrates detected by GC in all DHM batches, both before and after any heat treatment, were glucose and *myo*-inositol (Figure 2). The mean (SEM) glucose and *myo*-inositol concentrations in raw DHM samples were 272.1 (9.1) mg/L and 228.6 (13.6) mg/L, respectively. The duration but not the temperature of the HTST treatment had a statistically significant influence on the level of both compounds (repeated measures two-way ANOVA; *p* = 0.020 for glucose, and *p* = 0.037 for *myo*-inositol) (Table S1). Subsequently, HTST-treated samples were grouped according to the duration of the HTST treatment for further analysis (Figure 2). The glucose concentration in HTST-treated samples was similar to that found in raw DHM samples, with the exception of HTST-treated DHM for 25 s, which had a mean concentration approximately 28 mg/L lower than that of raw DHM (post hoc Dunnett’s test, *p* < 0.050; Figure 2). Conversely, the glucose content was higher (by approximately 22 mg/L) in HoP-treated DHM than in raw DHM (post hoc Dunnett’s test, *p* < 0.050; Figure 2). Furthermore, the *myo*-inositol concentration in DHM did not differ after any heat treatment from that in raw DHM (Figure 2).

### 3.3. Lipid Classes and FA Profile

The mean (SEM) values of the percentages of the main lipid classes and FA profile in DHM samples obtained from 10 unprocessed batches are shown in Table 2 and Table 3, respectively. In general, there was a wide variation in the levels of every lipid class and FA analyzed in the present study. TG represented 96.69% of the total lipids in the milk fat of DHM before heat treatment, while diacylglycerides (DG), which were the second most common class, accounted for only 2.78% (Table 2). Other lipid classes represented less than 0.5% of the total lipid count, including cholesterol plus free FAs (0.33%), monoacylglycerides (MG; 0.09%), polar lipids (PL; 0.06%), and cholesteryl esters (0.05%) (Table 2). Similar to other DHM components, the duration of the HTST treatment (but not the tested temperature) had a statistically significant impact on the percentage of most lipid classes (TG, DG, MG, and PL) (repeated measures two-way ANOVA, *p* < 0.050) (Table S2). Therefore, mean values of the percentages of each lipid class were grouped according to HTST treatment duration (ranging from 5 to 25 s) to compare the effect of HTST treatments (Table 2).

The following variations were observed in the lipid profile of HTST-treated DHM when compared to that of raw DHM (Table 2). First, the TG content was similar, and the DG fraction was 39% and 37% higher in HTST-treated DHM for the treatment times of 5 and 10 s, respectively, than in raw DHM and the MG fraction was nearly 50% lower than that in raw DHM after any HTST treatment. Second, the PL fraction in HTST-treated milk doubled in proportion, although no differences were noted in the levels of the individual PLs (Table 2). Third, the fraction containing cholesterol and FFAs was approximately one-third lower in HTST-heated DHM than in raw DHM after treatment for 5 and 20 s (Table 2). HoP also led to differences in the content of some lipid classes in relation to that of lipids in raw DHM; in particular, the MGs and the fraction of cholesterol plus free FA levels were 67% and 45% lower than in raw DHM, respectively. The cholesteryl ester fraction remained unchanged after all heat treatments (Table 2).

In relation to the FA profile in raw DHM samples, both SFAs and MUFAs were present in higher proportions (43% and 38%, respectively) than that of PUFAs (17%) (Table 3). The most abundant individual FAs were the MUFA oleic acid (C18:1 cis-9) that was present at 36%, the SFA palmitic acid (C16:0) at 23%, and the PUFA n-6 linoleic acid (C18:2 cis-9,12) at 16%. Other PUFAs found in DHM in lower amounts were α-linolenic acid (C18:3 cis-9,12,15; 0.24%), CLA (0.12%), ARA (C20:4 cis-5,8,11,14; 0.19%), and DHA (C22:6 cis-4,7,10,13,16,19; 0.08%) (Table 3).

Overall, the impact of the time and temperature variables of the HTST treatment in the FA profile was more linked to the duration of the treatment than to the temperature, although a significant interaction between the two factors was observed for most FAs (repeated measures two-way ANOVA, *p* < 0.050) (Table S3). HTST treatment for 15-25 s resulted in lower (4-6%) SFA levels and a higher (6-8%) proportion of PUFAs than in raw DHM, while MUFAs remained unaffected (Table 3). In contrast, HoP did not affect the mean values of SFAs, MUFAs, and PUFAs in relation to those of raw DHM. Table 3 shows the effect of the HTST treatment for 5 to 25 s or HoP on individual FAs. It should be noted that the percentage of CLA and DHA was higher (between 58 and 79% for CLA and 63 and 100% for DHA) in DHM after most HTST treatments, but not after HoP (Table 3).

### 3.4. Activity of BSSL

**Figure 3:**
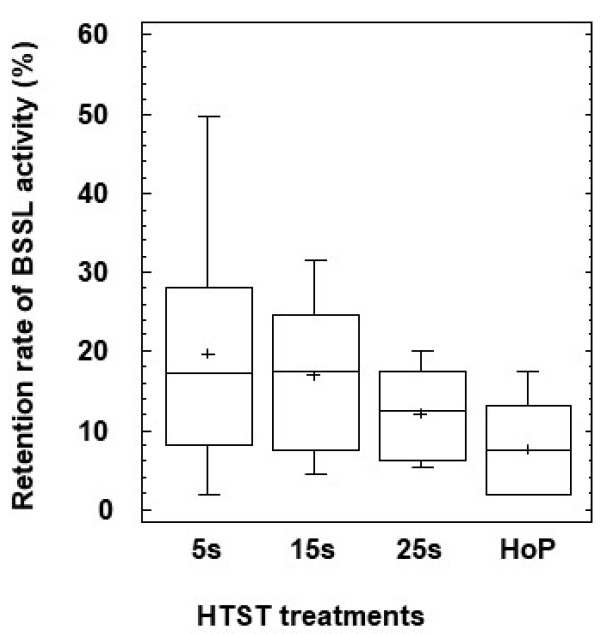
Retained bile salt stimulated lipase (BSSL) activity in DHM after HTST treatment for 5 (5 s), 15 (15 s), and 25 (25 s) seconds at 70 °C (*n* = 3) and 72 °C (*n* = 4) or HoP (62.5 °C, 30 min; HoP) (*n* = 7). BSSL, bile salt stimulated lipase; DHM, donor human milk; HoP, Holder pasteurization; HTST, high-temperature short-time.

The mean (SEM) value of BSSL activity in raw DHM (*n* = 7) was 9.26 (0.83) U/mL. BSSL activity was determined after HTST treatment at 70 °C (*n* = 3) and 72°C (*n* = 4) for 5, 15, and 25 s and after HoP. High variability in the inactivation of BSSL after HTST treatment for 5 s resulted in average retention rates of activity that ranged from 50% to 2% (Figure 3). The retention rates of BSSL activity were higher after HTST treatment for 5 s (mean value of 20% retention rate) than after HoP (7%) (repeated measures one-way ANOVA, *p* = 0.035) (Figure 3).

### 3.5. Vitamins

The contents of the most relevant vitamins in DHM samples (*n* = 5) before and after HTST treatment and HoP are presented in Table 4. A wide sample-to-sample variation was noted for both water- and fat-soluble vitamin concentrations in raw DHM. Among water-soluble vitamins, nicotinamide and vitamin B_2_ (riboflavin/flavin adenine dinucleotide) were the most abundant in raw DHM and were present at mean (SEM) concentrations of 501.8 (48.3) µg/L and 402.3 (26.9) µg/L, respectively. The levels of pyridoxal, thiamine and cyanocobalamin [mean (SEM) values of 91.0 (7.7) µg/L, 22.2 (2.9) µg/L and 0.5 (0.1) µg/L, respectively] were lower than those of nicotinamide and vitamin B_2_. Regarding fat-soluble vitamins, raw DHM contained 3.8 (0.2) mg/L α-tocopherol, 0.5 (0.1) mg/L γ-tocopherol, and 0.4 (0.1) mg/L vitamin A. Vitamins D_3_ and 25(OH)D_3_ were only present at low concentrations in raw DHM (mean (SEM) concentrations of 85.0 (14.8) µg/L and 26.6 (5.0) µg/L, respectively).

Neither of the two processing variables (temperature and duration of HTST treatment) had a statistically significant influence on the concentration of either water- or lipid-soluble vitamins (repeated measures two-way ANOVA, *p* > 0.05) (Table S4). HTST treatment did not affect the concentration of any vitamin that were present in raw DHM samples (Table 4). In contrast, the concentrations of pyridoxal and vitamin D_3_ were 12% lower and 47% higher in DHM after HoP than in raw DHM (repeated measures one-way ANOVA; *p* = 0.046 for pyridoxal and *p* = 0.036 for vitamin D3) (Table 4).

## 4. Discussion

In our study, we showed that the duration (5–25 s) of the HTST treatment had a higher impact on the nutrient composition of DHM than the temperature (70–75 °C). In general, the magnitude of the observed differences in nutrient content between HTST-treated and raw DHM was small, although statistically significant. This finding may indicate that HTST treatments have a modest but consistent effect on the nutrient composition of DHM, although the impact of the change in clinical practice remains unknown. In addition, our study demonstrated that HoP had a distinct impact on the nutritional composition of DHM when compared to HTST treatment.

BSSL activity was determined enzymatically. The height of the box indicates the IQR, the horizontal line in the box represents the median concentration value and the cross represents the mean concentration value. Values of retained BSSL activity after HTST treatment at 70 °C and 72 °C were grouped according to the duration of HTST treatment. Repeated measures one-way ANOVA tests were used to determine differences in the retention of BSSL activity between HTST-treated DHM for 5–15 s and HoP DHM. The asterisk indicates a significant difference in the pairwise comparison between HTST-treated DHM for 5 s and HoP DHM (post hoc Dunnett’s tests at the 95% confidence level.

Currently, most HMBs process DHM using HoP [3,11]. Several studies have addressed the effect of this type of pasteurization on milk macronutrients, reporting no differences in the protein and lactose content between raw and HoP-treated DHM [5]. In relation to fat, some authors found up to a 25% reduction in fat concentration in DHM after HoP when compared to that in untreated DHM [25], while others reported no significant differences between pre- and post-HoP-treated DHM [26]. In our study, the mean protein concentration in HoP-treated DHM was 0.2 g/L lower than that in raw DHM, but there was no difference in lactose and fat levels. This disparity among study results may reflect differences in sample preparation or analytical procedures. The macronutrient content of DHM was assessed in our study by using a mid-IR analyzer specifically validated for human milk analysis. On the other hand, the maximum average variations in lactose and fat content (>0.5 and <1.4 g/L, respectively) found in HTST-treated DHM compared to raw DHM in our analysis were statistically significant, but the differences were smaller than the natural batch-to-batch variation registered in unprocessed DHM [27]. Therefore, the inference might be that HTST treatments do not have a negative impact on the macronutrient composition of DHM. Moreover, the differences observed in lactose concentrations (∼1 g/dL) in our study depending on the analytical technique (mid-IR analyzer and GC) are most probably related to the inclusion of human milk oligosaccharides (HMOs) in the results provided by the mid-IR analyzer [28]. The values of total fat, protein and lactose in the samples analyzed in the present study before heat processing were consistent with those reported previously for DHM and similar to those described for fresh human milk [29]. A lower fat content in DHM than in freshly expressed human milk (3.0–4.0% vs. 3.5–4.5%) has been associated with the strong adherence of milk fat to container surfaces [29,30].

Our study also aimed to evaluate the impact of HTST treatments on the *myo*-inositol content of DHM because this sugar alcohol is strongly demanded by neonatal tissues, such as skeletal muscle and the epidermis, and it is found at high levels in neonatal blood and the newborn brain [31]. Our results indicate that neither HTST nor HoP treatments affected the *myo*-inositol content of raw DHM, and confirmed data from a previous study assessing the impact of HoP on DHM composition [32]. However, the *myo*-inositol concentration in mature milk (such as DHM) is lower than that found in colostrum and, probably, does not fulfill the requirements of the preterm infant [31,32].

Heat treatments (>70 °C) may denature some proteins in the milk fat globule membrane (MFGM), and this, combined with the freeze-thawing of milk, may favor lipolysis and the release of lipids into the aqueous phase [33]. The results presented in our study indicate that the TG fraction, the most abundant lipid class in DHM and the main energy source for preterm infants, was not modified after HTST or HoP treatments. In contrast, the content of MG was lower in both HTST- and HoP-treated DHM than in unprocessed DHM, which could partially explain the lower antimicrobial activity of pasteurized DHM compared to that of raw milk [34].

PLs are key components of the biological membrane enveloping milk fat globules whose structural and mechanical properties are important for milk fat digestion [35]. The PL/TG ratio in HTST-treated DHM in our study was twice that in raw DHM, which may indicate a size reduction in the milk fat globules [36]. The availability of a larger membrane surface on milk fat globules for the adsorption of digestive enzymes may improve fat absorption in preterm infants [37]. In contrast, the PL/TG ratio in HoP-treated DHM was equal to that of raw DHM.

The impact of HoP on the FA profile of DHM has been examined repeatedly, and, almost unanimously, all studies concluded that milk FA composition was unaffected by this heat treatment [6]. Our evaluation of individual FAs in HoP-treated DHM confirmed this fact. In contrast, our results indicated that HTST treatment for 15–25 s resulted in a lower content of SFAs (<4–7%) and a higher content of PUFAs (>6–8%) that in raw DHM. Overall, the most important variations in HTST-treated DHM that was processed for 25 s were found for CLA (158%), α- and γ-linolenic acids (142% and 155%, respectively), ARA (179%), and DHA (200%), which may indicate a higher lipid peroxidation in raw and HoP-treated DHM than in HTST-treated DHM samples during frozen storage. PUFAs, particularly long-chain PUFAs, are highly susceptible to peroxidation even during frozen storage [8], and our results denote that longer and more unsaturated PUFAs resulted in higher losses in raw and HoP-treated DHM. Alternatively, HTST treatment may preserve the antioxidant capacity of DHM, although the impact of different storage conditions of DHM on the antioxidant potential is currently unclear [8]. A higher PUFA content in DHM would be desirable for preterm nutrition because free FAs are readily absorbed in the immature gastrointestinal tract [37]. This finding may have biological and clinical relevance since preterm infants have a reduced bile pool and low pancreatic and lingual lipase activity [38]. The provision of LC-PUFAs by DHM to the preterm infant is relevant for the regulation of key physiological processes, as well as for the development and function of neural and immune tissues [39]. The higher CLA and DHA content in DHM after HTST treatment observed in our study may provide an additional benefit when compared to HoP.

Additionally, BSSL was not fully destroyed after the HTST process, resulting in 15–20% (mean values) retained BSSL activity. However, the high variability in BSSL residual activity after the shorter HTST treatments (5–15 s) highlights the importance of tight control of processing parameters to maximize the retention of this thermosensitive enzyme. Instead, BSSL was completely lost in DHM treated by HoP, as has been previously reported [9,40].

In our study, water- and fat-soluble vitamins were stable after both HTST treatment and HoP of DHM, with the exception of pyridoxal (11% lower in HoP than in raw DHM). In addition, processing conditions during HoP in our study may have facilitated the conversion of previtamin D_3_ into vitamin D_3_, as suggests the higher (145%) content of this vitamin in HoP-treated DHM compared than in unprocessed DHM. The relevance of these findings to clinical practice is still unclear given that there is no consensus about which is the most adequate intake of some vitamins, such as vitamin D or E, in preterm infants. Moreover, DHM is usually mature milk and does not guarantee appropriate vitamin coverage for preterm infants, for which it is often fortified [41,42].

High pressure processing (HPP) is a non-thermal technology widely applied now in the food industry and represents another promising alternative to HoP for treating DHM in human milk banks [12,43]. In HPP, food is subjected to pressures within the range of 100–1000 MPa for a short period of time (minutes). HPP treatment (200 MPa for 10 min, followed by an interval of 10 min, and 400 MPa for 10 min) at ambient temperature of DHM destroys efficiently milk vegetative bacteria but allows preserving the lipid profile, vitamins and some bioactive molecules, such as insulin, leptin, adiponectin, hepatocyte growth factor, IgG, BSSL, lysozyme and lactoferrin, when compared to HoP treatment [44,45,46,47]. The results of a recent study comparing HTST and HPP as potential alternatives to HoP to improve the quality of DHM indicated that both new processing technologies resulted in better DHM quality (regarding the protein profile) than classic HoP. However, both HTST and HPP treatments modified differently some bioactive molecules in DHM since, although secretory IgA was better preserved by HTST treatment, more lactoferrin activity was retained after HPP [48].

The results obtained in the present study are promising but also present a few limitations. First, some analytical determinations (BSSL activity and vitamin content) were not performed in all samples due to time and resource constraints. However, samples taken for these analyses were selected according to preliminary results to minimize the impact on the reported results. Furthermore, current benefits of HTST-pasteurized milk administration to preterm infants should be confirmed by additional clinical trials that are, in fact, currently in progress. For future studies, it should be taken into account that there are other factors that may influence the content of bioactive molecules in processed DHM, independently of the heat treatment, including the number of donors in the pooled sample and the storage conditions (i.e., refrigerated or frozen storage) of DHM samples before or after using any treatment [33,49,50].

HTST pasteurization at 72 °C for at least 10 s achieves the microbiological safety of DHM while ensuring a high retention of immunoglobulins, growth factors, and hormones [15,16]. The present study confirms that macronutrients, *myo*-inositol, lipid classes, and vitamins in DHM are preserved using the same equipment and processing variables as outlined in the above mentioned studies (Table 5). In addition, higher CLA and DHA levels together with higher retention of BSSL activity in HTST-treated DHM than in HoP-treated DHM could improve infant nutrition. In conclusion, our results indicate that this new HTST pasteurization system is an attractive alternative for the treatment of DHM in HMBs, particularly when considering that DHM is the feeding method of choice for preterm infants when OMM is not available [2,15,16].

## Figures and Tables

**Table 1:** Macronutrient composition of DHM before (raw) and after HTST treatment for 5 different durations of treatment, ranging from 5 to 25 s or HoP (62.5 °C, 30 min) (*n* = 10).

Nutrient	Raw	HTST					
		5 s	10 s	15 s	20 s	25 s	HoP
Lactose (g/L)	76.4 (0.3)	76.7 (0.3) *	76.9 (0.3) *	76.8 (0.3) *	76.8 (0.3) *	76.6 (0.3)	76.2 (0.3)
Fat (g/L)	34.7 (0.7)	34.8 (0.7)	34.5 (0.7)	34.4 (0.7)	33.9 (0.6) *	33.3 (0.6) *	35.0 (0.5)
Protein (g/L)	19.3 (0.2)	19.3 (0.2)	19.4 (0.2)	19.3 (0.2)	19.4 (0.2)	19.4 (0.2)	19.1 (0.2) *

* Asterisks indicate significant differences in pairwise comparisons between raw and heat-treated (HTST treatments for different durations or HoP) DHM (post hoc Dunnett’s tests at the 95% confidence level. Lactose, fat, and protein concentrations were determined by Fourier-transform mid-infrared spectroscopy and expressed as the mean (SEM) values. Data were grouped according to the duration of HTST treatment at 70, 72 and 75 °C before being analyzed using repeated measures one-way ANOVA including treatment (raw, HTST with different treatment durations and HoP) as fixed effects. HM, donor human milk; HoP, Holder pasteurization; HTST, high-temperature short-time.

**Table 2:** Percentage of lipid class levels in DHM before (raw) and after HTST processing for 5 different durations of treatment ranging from 5 to 25 s or HoP (62.5 °C, 30 min) (*n* = 10). ^1.^

Lipid Class	Raw	HTST					
		5 s	10 s	15 s	20 s	25 s	HoP
CE	0.05 (0.01)	0.03 (0.00)	0.04 (0.01)	0.03 (0.01)	0.04 (0.01)	0.04 (0.02)	0.03 (0.01)
CHOL + FFAs	0.33 (0.04)	0.22 (0.02) *	0.25 (0.03)	0.27 (0.03)	0.20 (0.04) *	0.28 (0.03)	0.18 (0.03) *
TG	96.69 (0.18)	95.71 (0.44)	95.73 (0.44)	95.79 (0.38)	96.16 (0.04)	96.39 (0.36)	97.26 (0.36)
DG	2.78 (0.19)	3.86 (0.42) *	3.80 (0.40) *	3.74 (0.36)	3.44 (0.41)	3.12 (0.32)	2.45 (0.33)
MG	0.09 (0.02)	0.05 (0.01) *	0.05 (0.01) *	0.05 (0.01) *	0.05 (0.01) *	0.05 (0.01) *	0.03 (0.01) *
∑PL	0.06 (0.01)	0.13 (0.11) *	0.14 (0.12) *	0.13 (0.01) *	0.12 (0.11) *	0.13 (0.12) *	0.06 (0.04)
PE	29.23 (0.72)	27.32 (1.19)	26.24 (1.46)	27.48 (2.58)	27.70 (1.84)	26.08 (1.79)	25.97 (1.43)
PI^2^	4.10 (0.39)	3.17 (0.11)	3.14 (0.21)	3.10 (0.64)	4.16 (0.71)	3.56 (0.16)	3.88 (0.26)
PS^2^	4.97 (1.04)	5.54 (0.09)	4.68 (0.94)	6.32 (1.41)	7.00 (1.61)	5.79 (0.48)	5.49 (0.39)
PC^2^	27.72 (0.81)	28.07 (1.65)	27.70 (1.47)	28.70 (3.33)	28.16 (1.77)	28.83 (0.63)	27.8 (0.78)
SM	33.97 (1.53)	35.90 (2.56)	38.25 (3.16)	34.39 (2.46)	32.97 (3.32)	35.73 (2.07)	36.86 (1.25)

^1^ Lipid classes were determined by HPLC-ELSD and expressed as the mean (SEM) values of percentages. Levels of the lipid classes CE, CHO + FFAs, TG, DG, MG, and PL were expressed as percentages of the total lipid DHM fraction. Levels of PE, PI, PS, PC, and SM were expressed as percentages of the total PL fraction. Data were grouped according to the duration of HTST treatment (at 70, 72 and 75 °C) before being analyzed using repeated measures one-way ANOVA including treatment (raw, HTST at different durations and HoP) as fixed effects. * Asterisks indicate significant differences in pairwise comparisons between raw and heat-treated (HTST treatment for different durations and HoP) DHM (post hoc Dunnett’s tests at the 95% confidence level).

**Table 3:** Percentage of fatty acid levels in DHM before (raw) and after HTST processing for 5 different durations ranging from 5 to 25 s or HoP (62.5°C, 30 min) (*n* = 10). ^1^

Fatty Acid	Raw	HTST					
		5 s	10 s	15 s	20 s	25 s	HoP
C8:0	0.15 (0.01)	0.16 (0.01)	0.14 (0.01)	0.14 (0.01)	0.15 (0.01)	0.14 (0.01)	0.19 (0.02)
C10:0	1.46 (0.06)	1.39 (0.07)	1.35 (0.06)	1.29 (0.04) *	1.29 (0.03) *	1.31 (0.04)	1.59 (0.09)
C12:0	6.28 (0.34)	5.77 (0.28)	5.72 (0.24) *	5.61 (0.16) *	5.46 (0.10) *	5.46 (0.15) *	6.46 (0.24)
C14:0	6.30 (0.38)	5.94 (0.33)	6.04 (0.33)	5.85 (0.28)	5.75 (0.25)	5.74 (0.27) *	6,19 (0.32)
C15:0	0.17 (0.01)	0.19 (0.01)	0.19 (0.02)	0.17 (0.01)	0.18 (0.01)	0.16 (0.02)	0.17 (0.01)
C16:0	22.65 (0.56)	21.46 (0.27)	21.67 (0.38)	21.74 (0.39)	21.70 (0.56)	21.02 (0.36) *	22.85 (0.46)
C17:0	0.15 (0.01)	0.19 (0.01) *	0.19 (0.01) *	0.17 (0.01)	0.18 (0.01) *	0.19 (0.01) *	0.14 (0.01)
C18:0	5.67 (0.22)	6.06 (0.25) *	6.13 (0.21) *	5.93 (0.16)	6.12 (0.27) *	6.23 (0.21) *	5.66 (0.18)
C20:0	0.15 (0.03)	0.23 (0.02) *	0.25 (0.04) *	0.22 (0.03) *	0.26 (0.02) *	0.24 (0.04) *	0.13 (0.06)
C16:1 *cis*-9	1.61 (0.07)	1.68 (0.08)	1.66 (0.09)	1.62 (0.07)	1.62 (0.11)	1.69 (0.08)	1.61 (0.08)
C18:1 *cis*-9	36.12 (0.90)	36.15 (0.86)	36.22 (1.11)	36.54 (0.90)	36.60 (0.95)	36.53 (0.79)	36.00 (0.84)
C18:1 *cis*-11	1.36 (0.07)	1.61 (0.04) *	1.57 (0.05) *	1.52 (0.04)	1.53 (0.10)	1.60 (0.03) *	1.35 (0.05)
C18:1 *trans*-11	0.11 (0.02)	0.21 (0.03) *	0.18 (0.03)	0.15 (0.03)	0.20 (0.04) *	0.22 (0.04) *	0.08 (0.02)
C18:2 *cis*-9,12	16.48 (0.27)	17.08 (0.35)	16.86 (0.39)	17.16 (0.34)	16.99 (0.49)	17.35 (0.35) *	16.44 (0.42)
CLA	0.12 (0.01)	0.20 (0.02) *	0.19 (0.02) *	0.17 (0.02)	0.20 (0.03) *	0.19 (0.03) *	0.10 (0.01)
C18:3 *cis*-9,12,15	0.24 (0.04)	0.31 (0.03)	0.31 (0.03)	0.33 (0.04)	0.32 (0.04) *	0.34 (0.04)	0.20 (0.02)
C18:3 *cis*-6,9,12	0.18 (0.03)	0.20 (0.03)	0.27 (0.02) *	0.23 (0.03)	0.27 (0.04) *	0.28 (0.04) *	0.12 (0.01)
ARA	0.19 (0.02)	0.30 (0.02) *	0.24 (0.04)	0.27 (0.02)	0.30 (0.04)*	0.34 (0.03) *	0.16 (0.02)
DHA	0.08 (0.01)	0.15 (0.02) *	0.14 (0.02) *	0.13 (0.02) *	0.14 (0.02)*	0.16 (0.02) *	0.06 (0.008)
SFAs	43.06 (1.04)	41.41 (0.66)	41.78 (0.82)	41.22 (0.57) *	41.18 (0.43) *	40.61 (0.49) *	43.46 (0.70)
MUFAs	37.59 (0.95)	37.97 (0.86)	38.15 (1.06)	38.20 (0.88)	38.33 (0.87)	38.34 (0.76)	37.43 (0.85)
PUFAs	17.29 (0.29)	18.32 (0.39)	18.08 (0.48)	18.33 (0.40) *	18.27 (0.63) *	18.72 (0.38) *	17.208 (0.44)
Total n-6 PUFAs	16.85 (0.27)	17.66 (0.36)	17.44 (0.43)	17.70 (0.37)	17.62 (0.56)	18.03 (0.36) *	16.71 (0.43)
Total n-3 PUFAs	0.32 (0.05)	0.46 (0.04) *	0.45 (0.05)	0.46 (0.06)	0.49 (0.04) *	0.50 (0.04) *	0.27 (0.02)

^1^ Fatty acids were determined by GC-MS and expressed as the mean (SEM) values of the percentage of total fatty acid methyl esters (FAMEs). Data were grouped according to the duration of HTST treatment (at 70, 72 and 75 °C) before being analyzed using repeated measures one-way ANOVA including treatment (raw, HTST at different durations and HoP) as fixed effects. * Asterisks indicate significant differences in pairwise comparisons between raw and heat-treated (HTST treatment for different durations or HoP) DHM (post hoc Dunnett’s tests at the 95% confidence level). ARA, arachidonic acid; CLA, conjugated linoleic acid; GC, gas chromatography; DHA, docosahexaenoic acid; DHM, donor human milk; HoP, Holder pasteurization; HTST, High-temperature short-time; MUFAs, monounsaturated fatty acids; PUFAs, polyunsaturated fatty acids; SFAs, saturated fatty acids.

**Table 4:** Vitamin concentrations in DHM before (raw) and after HTST treatment (processing at 70, 72 and 75 °C for 15, and 25 s) or HoP (62.5 °C, 30 min) (*n* = 5). ^1^

Vitamins	Raw	HTST Treatment	HoP
**Water–soluble vitamins**			
Thiamine (µg/L)	22.2 (2.9)	21.6 (3.1)	22.2 (1.6)
Riboflavin (µg/L)	33.2 (7.6)	34.3 (6.0)	37.5 (11.8)
FAD (µg/L)	369.1 (28.4)	427.3 (48.3)	427.1 (56.9)
Vitamin B_2_ (Riboflavin + FAD) (µg/L)	402.3 (26.9)	461.1 (41.3)	464.6 (54.9)
Nicotinamide (µg/L)	501.8 (48.3)	463.4 (40.7)	526.4 (34.5)
Pyridoxal (µg/L)	91.0 (7.7)	82.9 (5.8)	80.8 (4.8) *
Cyanocobalamin (µg/L)	0.5 (0.1)	0.5 (0.1)	0.6 (0.1)
**Lipid–soluble vitamins**			
Vitamin A (mg/L)	0.4 (0.1)	0.4 (0.1)	0.4 (0.1)
α-tocopherol (mg/L)	3.7 (0.2)	3.4 (0.2)	3.5 (0.2)
γ-tocopherol (mg/L)	0.5 (0.1)	0.5 (0.1)	0.5 (0.1)
Vitamin D_3_ (µg/L)	85.0 (14.8)	101.9 (18.1)	124.7 (28.4) *
Vitamin 25(OH)D_3_ (µg/L)	26.6 (5.0)	29.6 (5.2)	34.9 (4.9)

^1^ Vitamins were determined by HPLC and expressed as the mean (SEM) values. Data from all HTST-treated samples for 15 and 25 s at 70, 72 and 75 °C were grouped before being analyzed using repeated measures one-way ANOVA including treatment (raw, HTST treatment, and HoP) as fixed effects (*p* = 0.046 for pyridoxal and *p* = 0.036 for vitamin D3). * Asterisks indicate significant differences in pairwise comparisons between raw and HoP DHM (post hoc Dunnett’s tests at the 95% confidence level). DHM, donor human milk; FAD, flavin adenine dinucleotide; HoP, Holder pasteurization; HTST, high-temperature short-time.

**Table 5:** Comparison of the changes on DHM composition between HoP (62.5 °C, 30 min) and HTST treatment (72 °C for 15 s).

Nutrient ^1^	HTST	HoP
Lactose (by FT-MID)	↑ (0.05%) ^2^	-
Lactose (by GC)	-	-
Fat	-	-
Protein	-	↓ (1%)
Glucose	-	↑ (7%)
*myo*-Inositol	-	-
TG	-	-
DG	-	-
MG	↓ (45%)	↓ (67%)
∑PL	↑ (117%)	-
SFAs	↓ (4%)	-
MUFAs	-	-
PUFAs	↑ (6%)	-
CLA	↑ (42%)	-
ARA	-	-
DHA	↑ (63%)	-
Retention of BSSL activity	18%	7%
Thiamine, riboflavin, FAD, nicotinamide, cyanocobalamin	-	-
Pyridoxal	-	↓ (11%)
Vitamin A, α-tocopherol, γ-tocopherol, vitamin 25(OH)D_3_	-	-
Vitamin D_3_	-	↑ (47%)

^1^ Nutrient data were expressed as percentages compared to their value in raw milk. ^2^ ↑, increase; ↓, decrease; -, no change. ARA, arachidonic acid; BSSL, bile salt stimulated lipase; CLA, conjugated linoleic acid; DG, diacylglycerides; DHA, docosahexaenoic acid; DHM, donor human milk; FAD, flavin adenine dinucleotide; FT-MID, Fourier-transform mid-infrared spectroscopy; HoP, Holder pasteurization; HTST, high-temperature short-time; MG, monoacylglycerides; MUFAs, monounsaturated fatty acids; PL, polar lipids; PUFAs, polyunsaturated fatty acids; SFAs, saturated fatty acids; TG, triacylglycerides.

## Data Availability

The data presented in this study are available on request from the corresponding authors.

## Supplementary Materials

The following are available online at https://www.mdpi.com/2075-1729/11/2/114/s1, Figure S1. A. Flowchart depicting the milk pooling and heat processing of the production batches B. Analytical tests performed and number of samples analyzed in this study from the total number of production batches; Table S1. Effect of duration (time) and temperature of HTST treatment on lactose (GC), glucose, and myo-inositol content in DHM (*n* = 10); Table S2. Effect of duration (time) and temperature of HTST treatment on lipid class levels in DHM (*n* = 10); Table S3. Effect of duration (time) and temperature of HTST treatment on the FA profile of DHM (*n* = 10); Table S4. Effect of duration (time) and temperature of HTST treatment on the vitamin content of DHM (*n* = 4).
