# Supplementary material for: High-Temperature Short-Time and Holder Pasteurization of Donor Milk: Impact on Milk Composition
Source: Life (Basel). 2021 Feb 3;11(2):114. doi: 10.3390/life11020114 (PMC7913308; doi:10.3390/life11020114)
Supplement: Supplementary file 1 [file life-11-00114-s001.pdf]

Supplementary Material

High-temperature short-time treatment and Holder pasteurization of donor milk: impact on milk composition.

A

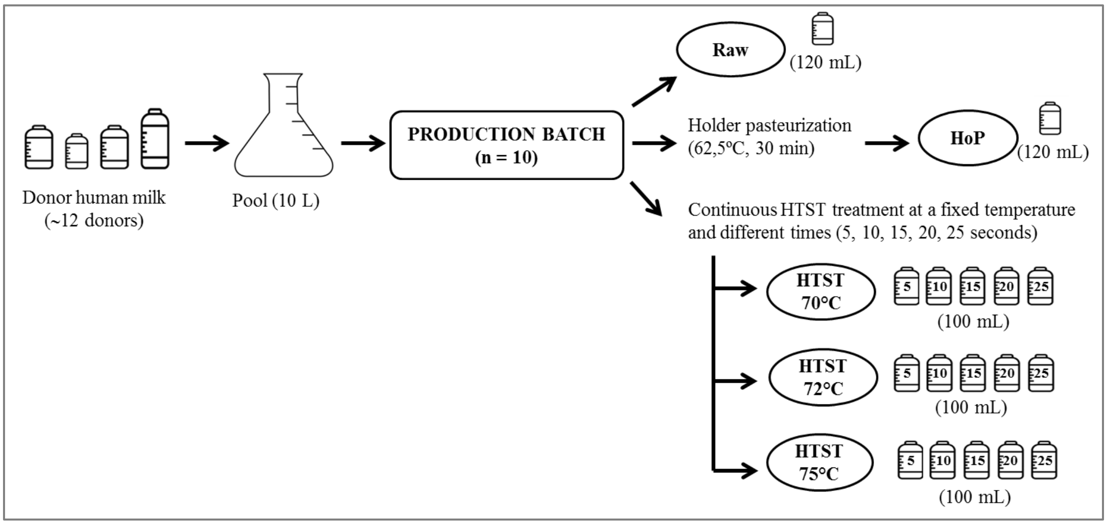

B

Analysis performed	Raw (*n* = 10)	HoP (*n* = 10)	HTST, 70 ºC (*n* = 3)	HTST, 72 ºC (*n* = 4)	HTST, 75 ºC (*n* = 3)
**Macronutrients** (FT-MID spectrometry)	10	10	3	4	3
**Lactose, glucose, *myo*-inositol** (GC)	10	10	3	4	3
**Lipid clases and FA profile** (HPLC, GC)	10	10	3	4	3
**BSSL activity** (enzymatic analysis)	7	7	3	4	-
**Vitamins** (UPLC-MS/MS, HPLC, UPLC-ESI-MS/MS)	7	7	1	2	2

Figure S1. (**A**). Flowchart depicting the milk pooling and heat processing of the production batches. An aliquot from all production batches (*n* = 10) was kept as control (raw milk) and a second aliquot was subjected to Holder pasteurization (62.5 ºC for 30 min). The rest of each production batch was HTST processed at a fixed temperature (70 ºC (*n* = 3), 72 ºC (*n* = 4), or 75 ºC (*n* = 3)) and samples were regularly taken at 5, 10, 15, 20, and 25 seconds as described by Escuder-Vieco et al. (1). (**B**). Analytical tests performed and number of samples analyzed in this study from the total number of production batches. HoP, Holder pasteurization; HTST, high-temperature short-time; FT-MID, Fourier-transform mid-infrared; GC, gas chromatography; HPLC, high performance liquid chromatography; UPLC-MS/MS, ultra performance liquid chromatography-tandem mass spectrometry; UPLC-ESI-MS/MS, UPLC-electrospray ionization tandem mass spectrometry.

(1) Escuder-Vieco D. Espinosa-Martos I. Rodríguez JM. Corzo N. Montilla A. Siegfried P. Pallás-Alonso CR. Fernández. L. High-Temperature Short-Time pasteurization system for donor milk in a human milk bank setting. Front Microbiol 2018;9:926.

Table S1. Effect of duration (time) and temperature of HTST treatment on lactose (GC), glucose, and *myo*-inositol content in DHM (n = 10)[1].

	Time		Temperature		Time × temperature interaction	
**Nutrient**	$F_{5,34}$	***P* value**	$F_{2,7}$	***P* value**	$F_{10,34}$	***P* value**
Lactose (GC)	1.07	0.396	4.22	0.063	0.51	0.870
Glucose	3.12	0.020	1.65	0.259	1.65	0.134
myo-inositol	2.69	0.037	1.21	0.355	2.18	0.045

[1]Each production batch was HTST processed at a fixed temperature (70 ºC (n = 3), 72 ºC (n = 4), or 75 ºC (n = 3)) and samples were regularly taken at 0, 5, 10, 15, 20, and 25 seconds. Lactose (GC), glucose, and *myo*-inositol concentrations were determined by GC. Data were analyzed using repeated measures two-way ANOVA tests, including time, temperature, and their interaction as fixed effects; degrees of freedom between and within groups are given as subscripts to F.

DHM, donor human milk; GC, gas chromatography; HTST, high-temperature short-time.

Table S2. Effect of duration (time) and temperature of HTST treatment on lipid class levels in DHM (n = 10)[1].

	Time		Temperature		Time × temperature interaction	
**Lipid class**	$F_{5,35}$	P **value**	$F_{2,7}$	P **value**	$F_{10,35}$	P **value**
CE	0.79	0.564	17.15	0.002	6.01	<0.001
CHOL + FFA	1.58	0.191	0.90	0.448	0.55	0.846
TG	4.90	0.002	3.78	0.077	0.59	0.810
DG	5.92	<0.001	3.67	0.081	0.62	0.788
MG	6.01	<0.001	4.65	0.052	0.52	0.864
∑PL	5.29	0.001	6.42	0.026	0.87	0.570
PE	1.17	0.345	0.22	0.807	1.33	0.253
PI	2.69	0.037	0.01	0.987	3.00	0.008
PS	1.17	0.344	0.88	0.455	1.56	0.159
PC	0.78	0.569	4.82	0.048	1.20	0.326
SM	1.88	0.124	3.60	0.084	1.99	0.066

[1] Each production batch was HTST processed at a fixed temperature (70 ºC (n = 3), 72 ºC (n = 4), or 75 ºC (n = 3)) and samples were regularly taken at 0, 5, 10, 15, 20, and 25 seconds. Lipid classes were determined by HPLC-ELSD and their mean (SEM) values are presented in Table 2. Data were analyzed using repeated measures two-way ANOVA tests, including time, temperature, and their interaction as fixed effects; degrees of freedom between and within groups are given as subscripts to F.

CE, cholesteryl ester; CHOL, cholesterol; DG, diacylglycerol; DHM, donor human milk; FFA, free fatty acid; HPLC, high-performance liquid chromatography; HTST, high-temperature short-time; MG, monoacylglycerol; PC, phosphatidylcholine; PE, phosphatidylethanolamine; PI, phosphatidylinositol; PL, polar lipids; PS, phosphatidylserine; SM, sphingomyelin; TG, triacylglycerol.

Table S3. Effect of duration (time) and temperature of HTST treatment on the FA profile of DHM (n = 10)[1].

	**Time**		**Temperature**		**Time × Temperature Interaction**	
**Fatty acid**	$F_{5,35}$	***P* value**	$F_{2,7}$	***P* value**	$F_{10,35}$	***P* value**
C8:0	1.40	0.249	1.82	0.230	1.93	0.074
C10:0	5.24	0.001	4.57	0.054	1.80	0.098
C12:0	8.61	<0.001	10.64	0.008	3.73	0.002
C14:0	6.89	<0.001	4.80	0.049	3.04	0.007
C15:0	1.51	0.211	3.39	0.094	1.15	0.359
C16:0	3.26	0.016	0.63	0.562	2.77	0.012
C17:0	6.41	<0.001	13.82	0.004	3.21	0.005
C18:0	4.11	0.005	0.28	0.766	1.65	0.133
C20:0	2.81	0.031	4.50	0.055	4.55	<0.001
C16:1 *cis*-9	0.41	0.836	1.33	0.325	0.94	0.513
C18:1 *cis*-9	0.29	0.914	1.89	0.221	0.22	0.993
C18:1 *cis*-11	3.28	0.016	1.05	0.399	2.28	0.036
C18:1 *trans*-11	3.74	0.008	4.00	0.070	2.56	0.019
C18:2 *cis*-9,12	3.64	0.009	0.38	0.696	3.43	0.003
CLA	11.24	<0.001	3.73	0.079	5.61	<0.001
C18:3 *cis*-6,9,12	9.59	<0.001	0.29	0.759	6.67	<0.001
C18:3 *cis*-9,12,15	1.95	0.110	4.36	0.059	1.98	0.067
ARA	7.92	<0.001	9.76	0.010	7.17	<0.001
DHA	9.23	<0.001	5.58	0.036	5.21	<0.001
SFA	4.71	0.002	1.17	0.365	3.17	0.005
MUFA	0.71	0.622	1.71	0.249	0.26	0.985
PUFA	5.44	<0.001	0.52	0.618	4.60	<0.001

[1] Each production batch was HTST processed at a fixed temperature (70ºC (n = 3), 72ºC (n = 4), or 75ºC (n = 3)) and samples were regularly taken at 0, 5, 10, 15, 20, and 25 seconds. Fatty acids were determined by GC and the mean (SEM) values of the percentage of total fatty acid methyl esters (FAMEs) are presented in Table 3. Data were analyzed using repeated measures two-way ANOVA tests, including time, temperature, and their interaction as fixed effects; degrees of freedom between and within groups are given as subscripts to F.

ARA, arachidonic acid; CLA, conjugated linoleic acid; GC, gas chromatography; DHA, docosahexaenoic acid; DHM, donor human milk; HTST, high-temperature short-time; MUFA, monounsaturated fatty acid; PUFA, polyunsaturated fatty acid; SFA, saturated fatty acid.

Table S4. Effect of duration (time) and temperature of HTST treatment on the vitamin content of DHM (*n* = 4)[1].

	Time		Temperature		Time × Temperature Interaction	
**Vitamin**	$F_{5,35}$	***P* value**	$F_{2,7}$	***P* value**	$F_{10,35}$	***P* value**
Thiamine	0.00	0.967	2.87	0.169	1.64	0.301
Riboflavin	2.51	0.254	1.10	0.416	0.21	0.818
Flavin-adenine- dinucleotide	0.13	0.749	2.33	0.214	0.30	0.753
Vitamin B_2 (riboflavin+FAD)	0.04	0.855	2.20	0.226	0.27	0.776
Nicotinamide	2.76	0.239	2.05	0.243	0.03	0.971
Pyridoxal	0.13	0.751	1.63	0.303	0.07	0.929
Cyanocobalamin	0.03	0.873	1.18	0.395	0.50	0.639
Vitamin A	0.06	0.824	1.58	0.313	1.68	0.296
α-tocopherol	0.08	0.799	1.51	0.325	0.92	0.468
γ-tocopherol	1.00	0.423	0.63	0.576	1.06	0.428
Vitamin D_3	4.22	0.176	3.52	0.131	1.89	0.265
Vitamin 25(OH)D_3	0.82	0.461	0.14	0.874	1.03	0.435

[1]Each production batch was HTST processed at a fixed temperature (72 ºC (*n* = 2). or 75 ºC (*n* = 2)) and samples were taken at 15, and 25 seconds. Vitamins were determined by HPLC and the mean (SEM) values are presented in Table 4. Data were analyzed using repeated measures two-way ANOVA tests including time, temperature, and their interaction as fixed effects; degrees of freedom between and within groups are given as subscripts to *F*.

DHM. donor human milk; FAD. flavin adenine dinucleotide; HTST. high-temperature short-time.
